# Volume of sputum to detect acid-fast *bacilli* as a measure of quality for the diagnosis of pulmonary tuberculosis at the Dr George Mukhari Hospital, South Africa

**DOI:** 10.4102/phcfm.v3i1.240

**Published:** 2011-10-14

**Authors:** Iqbal Rashid, Langalibalele H. Mabuza, Indiran Govender, Deidre Pretorius

**Affiliations:** 1 Department of Family Medicine and Primary Health Care, University of Limpopo (Medunsa Campus), South Africa

## Abstract

**Background:**

Optimum sputum results for acid-fast *bacilli* (AFB) microscopy are linked to a sputum quantity of at least 5.0 mL. This study was aimed at establishing the effect of sputum quantity in the pick-up rate of AFB microscopy by comparing sputum samples of 5.0 mL and 2.0 mL.

**Methods:**

An analytical cross-sectional study was carried out at the Dr George Mukhari Hospital (DGMH) in Pretoria, South Africa, from 05 January 2007 to 04 January 2008. Two sputum samples, 5.0 mL and 2.0 mL, were collected from each of the 330 adult PTB (pulmonary tuberculosis) suspects. Fluorescence microscopy was used in the sputum analysis. The yield through microscopy of the 2.0 mL specimen versus the 5.0 mL specimen was compared and analysed, using culture results as the gold standard.

**Results:**

From a sample of 330 specimens, 77 tested AFB positive on microscopy. In the 5.0 mL samples, the sensitivity was 76.6% (95% CI, 66.0% – 84.7%), specificity 99.6% (95% CI 97.8% – 99.9%), positive predictive value (*PV+*) 98.3% (95% CI 91.1% – 99.7%), negative predictive value (*PV-*) 93.3% (95% CI 89.7% – 95.7%), the likelihood ratio (LR) for a positive microscopy 192 and the LR for a negative test was 0.23. In the 2.0 mL specimens, the sensitivity was 75.3% (95% CI 64.6% – 83.6%), specificity 99.2% (95% CI 97.1% – 99.8%), positive predictive value (*PV+*) 96.7% (95% CI 88.6% – 99.1%), negative predictive value (*PV-*) 93.0% (95% CI 89.3% – 95.4%), the LR for a positive microscopy was 94 and 0.25 for a negative microscopy. There was a statistically significant association (*p*-value < 0.001) between the microscopy and culture tests in both the 5.0 mL and the 2.0 mL specimen categories. The strength of association between the microscopy and culture, as indicated by the kappa test was 0.83 and 0.81 in the 5.0 mL and 2.0 mL categories, respectively.

**Conclusion:**

Compared to the 2.0 mL specimen category, the yield for AFB microscopy in the 5.0 mL specimen category was consistently superior, as indicated by the higher sensitivity, specificity, predictive values and the likelihood ratios in the 5.0 mL specimen category. It is recommended that sputum specimen collection for AFB microscopy should aim for a minimum volume of 5.0 mL.

## Introduction

### Setting

Globally, an estimated 8 million new cases of tuberculosis occur annually, mostly in developing countries.^1, 2^ According to the World Health Organisation (WHO) 2010-report, the estimates of the global burden of disease caused by TB (tuberculosis) in 2009 were 9.4 million incident cases and 14 million prevalent cases.^3^ In South Africa the control of TB continues to be a major concern.^4^

The Ziehl Nielsen (ZN) staining procedure is the cornerstone of demonstrating the acid-fastness of the bacteria. Prompt diagnosis of TB plays a pivotal role in therapy outcome. However, some cases suspected of pulmonary tuberculosis on clinical grounds, are not confirmed bacteriologically because of a patient having a dry cough or inadequate sputum (< 2.0 mL). This results in false negative sputa in about 25% – 50% of cases of pulmonary tuberculosis.^5^

### Contribution to the field

Adequate sputum is required for a diagnostic yield in AFB detection. Researchers have suggested the use of 5.0 mL as adequate sputum for AFB detection in the diagnosis of PTB (Pulmonary tuberculosis).^6, 7, 8^ In this regard, the study became necessary because, at the time of the study, the Level One wards at the hospital did not have a policy relating to a recommended sputum quantity. We conducted this study to compare the yield of a 5.0 mL versus a 2.0 mL specimen.

## Ethical considerations

Ethical clearance to conduct the study was obtained from the Medunsa Research Ethics Committee (MREC) of the University of Limpopo (Clearance Certificate Number: MREC/M/50/2009).

## Method

### Design

From 05 January 2007 to 04 January 2008, a cross-sectional descriptive study was conducted at the Dr George Mukhari Hospital, Pretoria, to determine if the volume of sputum affects AFB microscopy yield. Each patient was requested to cough up two sputum samples at least 2 hours apart in the early morning. There is no consensus or evidence on the acceptable time lag (e.g. 2 hours versus 24 hours apart) between sputum sample collections for a better yield in sputum smear microscopy. The research assistant (a nurse) supervised the sputum collection. Each patient was requested to cough up sputum into the specimen bottle until the sputum quantity reached the labelled mark on the bottle, 2.0 mL or 5.0 mL. When necessary, a patient was requested to cough up additional sputum to fill up the specimen bottle to the required mark. On an alternating basis, one patient was requested to start filling up the 5.0 mL green-labelled specimen bottle, followed by the 2.0 mL red-labelled specimen bottle at least 2 hours apart. The next patient was requested to reverse the order, starting with the 2.0 mL, then the 5.0 mL specimen bottle. This was done to ensure an even distribution in the order of sputum quantity collection to minimise a sputum collection bias.

### Materials

The number of PTB suspects per annum seen at DGMH (primary health care) in 2007 was 2348.^9^ During the period of data collection, sputum samples were collected randomly from TB suspects willing to participate in the study. Clinical suspicion of TB in patients was based on at least three of the following criteria:

- a persistent cough for a period longer than 2 weeks
- night sweats
- fever
- loss of appetite
- weight-loss
- malaise
- chest pain
- shortness of breath
- a positive history of contact with an infectious TB patient.

Consenting patients provided sputa in sterile containers at room temperature.

### Patients

Only patients above the age of 18 who consented to participate were included in the study. Using the SPSS 17.0 for Windows computer software, the representative sample size computed at 95% confidence level and 5% confidence interval (CI), was 330 patients. Randomisation was achieved by selecting every seventh PTB suspect admitted to Level One wards for inclusion in the study. If a particular patient declined participation, the next consenting patient was included, to be followed by another seventh patient. Patients too ill to participate were excluded from the study.

Two sputum samples, one of 5.0 mL and the other of 2.0 mL, were collected from each adult PTB suspect. Patients were advised on how to enhance sputum expectoration. Sputum samples were then taken to the laboratory within 2 hours of collection. The sputum was analysed with the aid of *auramine* staining and fluorescence microscopy as previously described.^10^

Recruited patients were educated on the importance of producing a good sputum sample (not saliva or nasal discharge) and it was explained to them that two specimens with different quantities were required. The patient was then shown and instructed on how to enhance sputum expectoration. Sputum collection took place in an open area under direct supervision of one of the researchers to prevent infection of the researchers and other patients.

This following section does not describe data collection but specimen collection and laboratory methods.

### Specimen collection and laboratory methods

The patient was given two sterile sputum containers, one labelled with a green marker and the other labelled with a red marker. The green-labelled container was for the collection of 5.0 mL sputum whilst the red-labelled container was for 2.0 mL sputum. The level of the volume required was clearly marked on each specimen container to guide patients as to the sputum quantity required. Both the 2.0 mL and the 5.0 mL sputum containers were subsequently sent for microscopy and culture. Each specimen was given a different laboratory number and all specimens were thus treated separately as though they were from different patients. Consequently, two laboratory analysis requisition forms were completed for each patient. Each specimen and attached requisition form were put into specimen plastics, sealed and immediately taken to the laboratory. If there was an anticipated delay of more than 2 hours in transporting the specimen to the laboratory, the specimen was stored in a refrigerator at 4 °C.

Only sputum samples collected on the first morning were used in the study. Sputum samples collected on subsequent mornings or days, as required by the national tuberculosis programme for diagnosis, were not included in the study. The culture was grown according to standard procedures by the National Health Laboratory Services (NHLS) of South Africa, using the Mycobacteria Growth Indicator Tube 7 mL (MGIT) method. Culture tubes were entered into the BACTEC MGIT instrument and continuously incubated at 37 °C and monitored every 60 minutes for increasing fluorescence. Analysis of the fluorescence was used to determine if the tube was instrument-positive, that is, if the test sample contained viable organisms. Culture tubes which remained negative for a minimum of 42 days were regarded as negative.

## Analysing

The statistical software mentioned above was used for data entry and analysis. Descriptive statistics, frequencies and percentages were calculated. Two-by-two tables were drawn to determine the relationship between the 5.0 mL and 2.0 mL specimens as compared to the culture which was the gold standard test. For statistical significance, the *p*-value was determined whilst *kappa* was used to measure the strength of agreement. The sensitivity, specificity, positive and negative predictive values, and likelihood ratios for a positive and negative microscopy results of sputum samples were calculated to determine the relationship between AFB microscopy and culture tests.

## Results

The majority of the patients, 110 (32.1%), were aged 18–29, 95 (28.8%) were aged 30–40, 68 (20.6%) were 41–51 years old, 38 (11.5%) were aged 52–62, 21 (6.4%) were 63–73 years old and 2 (0.6%) were aged 74–94.

Of the 330 patients, 146 (44.2%) were male and 184 (55.8%) were female. The prevalence of AFB positive sputum results in the 2.0 mL samples was 17.6% (58/330) and 17.9% (59/330) in the 5.0 mL samples.

Sixty patients (18.2%) were positive and 270 (81.8%) were negative for the AFB test in the 5.0 mL category, whilst 58 (17.6%) were positive and 272 (82.4%) were negative for the AFB test in the 2.0 mL category. The gold standard AFB culture results revealed 77 (23.3%) as positive and 53 (76.7%) as negative sputa.

In the 5.0 mL sputum samples, out of the total of 77 specimens that were positive according to the gold standard culture test, 59 tested positive on AFB microscopy, resulting in a sensitivity of 76.6% (59/77), (95% CI 66.0% – 84.7%), (Table 1). Meanwhile, according to the culture test, 253 specimens tested negative whilst 252 tested negative by microscopy, missing one specimen (false negative), resulting in a specificity of 99.6% (252/253), (95% CI 97.8% – 99.9%). The positive predictive value (*PV+*) was 98.3% (59/60), (95% CI 91.1% – 99.7%) whilst the negative predictive value (*PV-*) was 93.3% (252/270), (95% CI 89.7% – 95.7%). The likelihood ratio (LR) for a positive microscopy in the 5.0 mL volume sputa was 192 [76.6 ÷ (100 - 99.6)], whilst the LR for a negative test was 0.23 [(100 - 76.6) ÷ 99.6].

**FIGURE 1:**
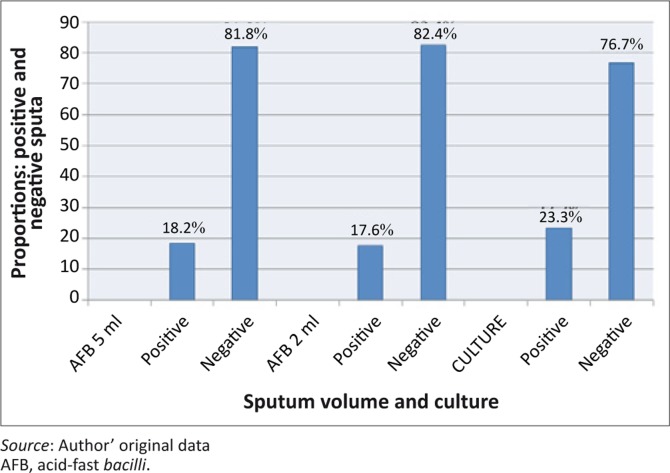
Distribution between AFB (5.0 mL), AFB (2.0 mL) and Culture smear.

**TABLE 1:** Relationship between acid-fast *bacilli* (5.0 mL) and culture for diagnosis of pulmonary tuberculosis (*N* = 330).

AFB (5 mL)	Culture Pulmonary TB		Total

	(*PV+*)	(*PV-*)	
(*PV+*)	59	1	60
(*PV-*)	18	252	270

**Total**	**77**	**253**	**330**

*Source*: Author’ original data *N*, Number of patients with probable PTB; (*PV+*), predictive value positive; (*PV-*), predictive value negative; AFB, acid-fast *bacilli*; PTB, Pulmonary tuberculosis.

Table 2 demonstrates that out of the 77 sputum smears (from the 2.0 mL specimens) which tested positive by culture, 58 were picked up by microscopy, resulting in a sensitivity of 75.3% (58/77), (95% CI 64.6% – 83.6%). Meanwhile, out of the total of 253 specimens that were negative according to the culture test, 251 tested negative by microscopy and two were missed (false positives), resulting in a specificity of 99.2% (251 253), (95% CI 97.1% – 99.8%). The positive predictive value (*PV+*) was 96.7% (58/60), (95% CI 88.6% – 99.1%) whilst the negative predictive value (*PV-*) was 93.0% (251/270), (95% CI 89.3% – 95.4%). The likelihood ration (LR) for a positive microscopy in the 2.0 mL specimens was 94 [75.3 ÷ (100 - 99.2)], whilst the LR for a negative microscopy was 0.25 [(100 - 75.3) ÷ 99.2].

**TABLE 2:** Relationship between acid-fast *bacilli* (2.0 mL) and culture for diagnosis of pulmonary tuberculosis (*N* = 330).

AFB (2.0 mL)	Culture Pulmonary TB		Total

	(*PV+*)	(*PV-*)	
(*PV+*)	58	2	60
(*PV-*)	19	251	270

**Total**	**77**	**253**	**330**

*Source*: Author’ original data *N*, Number of patients with probable PTB; (*PV+*), predictive value positive; (*PV-*), predictive value negative; AFB, acid-fast *bacilli*; PTB, Pulmonary tuberculosis.

The 5.0 mL sputum smear test gave false negative results in 23.4% (18/77), (95% CI 15.3% – 34.0%) patients whilst the 2.0 mL sputum smear test gave a false negative value of 24.7% (19/77), (95% CI 16.4% – 35.4%). There were 0.4% (1/253), (95% CI 0.07% – 2.2%) false positive results encountered for 5.0 mL and 0.8% (2/253), (95% CI 0.2% – 2.8%) for the 2.0 mL sample.

## Discussion

The majority of the patients (60.9%) were between the ages of 18 and 40 years, of whom 53.7% were 18–29 years old. The incidence in the 18–40 age group in this study was slightly higher than a similar study conducted in Mexico (52%).^11^ We were focussing on PTB suspects on clinical grounds, hence a wider scope, whereas their focus was mainly on radiological changes. The fact that TB suspects are in the young adulthood age-group (hence breadwinners), has economic implications for the society. This places the onus on the clinician to properly diagnose each TB suspect by using the sputum quantity with a better yield, to enable early therapeutic intervention.

The prevalence rate of the AFB positive sputa by microscopy was virtually similar in the 5.0 mL versus the 2.0 mL sputum specimens (17.9% versus 17.6%, respectively). The smear sensitivity rate of 76.6% obtained using the 5.0 mL sputum specimen, and 75.3% obtained using the 2.0 mL specimen, from patients suspected of having TB in this study, was in keeping with the upper limit of the range of sensitivity for smear microscopy reported by Foulds and O'Brien to vary from 30% to more than 70%.^12^ Matee et al., in a study conducted in the Muhimbili University in Dar es Salaam, Tanzania, sought to determine the diagnostic accuracy of sputum microscopy for active case findings of HIV-associated PTB using TB culture as the reference standard. No mention is made of the quantity of sputum used. However, their overall sensitivity and specificity of sputum microscopy was 61.8% and 99.7%, respectively,^13^ which were lower than found in this study. This could be ascribable to the relatively smaller sample of culture positive sputum results in this study compared to their sample (77 versus 212).

In a study conducted by Warren et al. over two periods, 1992–1993 and 1996–1999, the yield using a minimum of 5.0 mL sputum specimen for AFB diagnosis was found to increase sensitivity for *Mycobacterium tuberculosis* (MTB), thus accelerating treatment of TB. In the first period, the acid-fast staining was carried out using sputum specimens regardless of volume. In that way, a sensitivity of 72.5% was obtained, compared to 92.0% for sputum specimens of volumes ≥ 5.0 mL during the second period.^14^ The better yield of the sputum specimens with the minimum of 5.0 mL tallied with our study even though the methodologies used in the two studies differed. The relatively low sensitivity in our study could be attributable to the comparatively smaller sample size and the high prevalence of HIV seropositive patients (65.2%) in our Level One wards, because there is an association between decreased host immunity and reduced sputum smear positivity leading to an increase in false negative smears.^15^

In contrast with the likelihood ratio for a negative microscopy test that was almost similar in both the 5.0 mL and 2.0 mL sputum samples, the likelihood ratio for a positive microscopy in the 5.0 mL sample was almost double that in the 2.0 mL samples (192 versus 94, respectively), clearly in favour of the 5.0 mL sputum sample for AFB microscopy. Clinically, this finding indicates that by using a 5.0 mL specimen, the probability of finding a false positive in a truly negative result is almost 50% lower than by using the 2.0 mL specimen. This finding was corroborated by the relatively higher positive and negative predictive values in the 5.0 mL specimen category, compared to the 2.0 mL category. The difference is of clinical importance because the diagnostic value of a test depends on its positive and negative predictive values, which vary with the prevalence of the disease in a given community.^16^

As mentioned above, the smear microscopy used in this study was based on fluorescence microscopy of concentrated sputum sediments following centrifugation and is commonly considered to have superior sensitivity compared with conventional light microscopy.^17^ The false negative results were slightly lower in the 5.0 mL specimens compared to the 2.0 mL specimens (23.4% versus 24.7%, respectively). Furthermore, the false positive results in the 5.0 mL specimens were 50% lower than those in the 2.0 mL samples (0.4% versus 0.8%, respectively). An increase in false positive results reduces the specificity of a test by increasing the denominator of specificity.^18^ The clinical implication of an increase in false positives is the over-diagnosis of a condition with unnecessary commitment of resources on subjects who do not need the resources.

Supervised sputum collection by a health care professional during sputum collection is an important factor in obtaining quality sputum samples. Improving sputum quality has been shown to improve the yield of sputum microscopy.^14^ Therefore, the best use of limited resources for the detection of smear-positive PTB cases would be to improve the quality of self-expectorated sputum collection and microscopy. Only supervised sputum collection was used in our study, we did not use augmented and invasive sputum collection techniques as recommended in other studies.^19, 20^ However, PTB suspects who are unable to produce sputum, should be followed-up regularly or even admitted to a health-care facility if possible. The inability to produce sputum is common in immunocompromised patients.^21^ There was a high prevalence of sero-positive patients in our Level One wards which led the researchers to the conclusion that every effort should be attempted to obtain adequate sputum from every suspected PTB sufferer. To enhance sputum collection, various methods are available, from sputum induction and physiotherapy, to more invasive procedures like bronchoalveolar lavage.^11, 22^

It has been established from the literature that patient education, allied health worker training and involvement of the community are also of value in a TB programme.^18^ Sputum collection should be done preferably in the early morning, in a secluded and well ventilated environment so that it does not pose a threat of infection to others. Supervised sputum collection combined with patient instruction, is of great value in obtaining a good quality sputum specimen (not mere saliva), as demonstrated by Sakundarno et al. in a study to determine factors associated with insufficient quality of sputum collected for PTB diagnosis in Indonesia.^23^ They deduced that, in their district, only a few PTB suspects (45%) provided samples of good colour, viscosity and volume (3.0 mL). One of the reasons identified for this result was poor patient education by health care workers on how to collect sputum. In our study patients were guided on the correct way to provide sputum.

Sputum samples that were collected for this study were kept less than 1 hour after collection (en route to the laboratory), to avoid contamination with other micro-organisms from possible excessive specimen handling in the ward. Studies have shown that sputum specimens for AFB detection could be preserved safely in the refrigerator (4 °C) for up to 8 weeks without affecting the sputum-smear positivity result.^24, 25^ It has also been shown that there is always the element of specimen container contamination in the wards which could lead to specimen contamination before the specimen reaches the laboratory.^26, 27^ Nevertheless, in spite of the precautions, 8.5% of the specimens in our study tested positive for mycobacteria other than tuberculosis (MOTT) by culture.

Direct microscopy results tend to display variability depending on the microscopic field used, and the number of *bacilli* in a specific field as well as the level of expertise of the microscopist. Comparing direct sputum microscopy and the NALC-NaOH (N-acetyl-L-cysteine-sodium hydroxide) method, Farnia et al. established that the NSLC-NaOH method was more reliable. They found the sensitivity and specificity of NALC-NaOH method to be 83% and 97% respectively, which was higher than that of direct sputum microscopy which stood respectively at 46% and 90%.^28^ The NALC-NaOH method was used in this study. This method, however, is not performed in countries with limited resources. Errors by the microscopists in examining sputum smear slides could also have affected our study results, thereby increasing the number of false negative smears. A study has shown that implementation of proficiency testing of microscopists and the introduction of a rechecking system for external quality assurance could help in evaluating diagnosis.^29^ The process is labour intensive, however, especially in countries with a high TB burden. The limitation of this study was the relatively small sample size.

## Conclusion

This study has indicated a tendency towards superiority in the AFB microscopy results yield of the 5.0 mL specimen category compared to the 2.0 mL category as indicated by the relatively higher sensitivity, specificity and predictive values. With regard to the likelihood ratio (LR), given a positive microscopy with a 5.0 mL specimen, the ratio of a truly positive to a truly negative ratio is 192:1, that is, it is 192 times more likely to be truly positive than truly negative. With a 2.0 mL specimen, the LR is 94:1. Therefore with a 5.0 mL specimen there is a greater chance (more than twice better) of finding a truly positive result. From this point of view, the 5.0 mL specimen is to be recommended.
